# Should we spend less or control a greater percentage of hypertensive patients?

**DOI:** 10.1590/S1516-31802002000400001

**Published:** 2002-07-07

**Authors:** Décio Mion, Katia Coelho Ortega

Adherence to hypertension treatment forms a major challenge because, despite the relatively simple and cheap diagnosis combined with efficacious treatments, the majority of patients do not adhere to the treatment and do not present controlled arterial pressure. A large number of factors have been related to low adherence rates.^1^ When we asked patients in our service (n = 353) regarding the causes of low adherence, we obtained the following results: a) medicines - high cost (89%), taking them several times per day (67%) and collateral effects (54%); b) disease - not knowing how serious it is (50%) and absence of symptoms (36%); c) knowledge and beliefs - only taking the medicine when the pressure is high (83%), not taking care of health (80%), forgetting to take the medicines (75%), not knowing its chronic nature (70%) and complications of the disease (70%); and d) doctor-patient relationship - remaining unconvinced about undertaking treatment (51%) and inadequate relationship (20%).^2^ Thus, the cost of treatment is a very important cause of low adherence.

In a excellent study, through a cross-sectional population-based study in the urban area of Pelotas (southern Brazil), Costa et al. described the healthcare costs for the treatment of hypertension and examined the cost-effectiveness of different classes of antihypertensives. The authors interviewed 1,968 participants in their homes and observed that 23.5% had high pressure (≤ 160/95 mmHg) or were making use of antihypertensives. Although the authors justified the use of these values so as to reduce the potential for bias in pressure measurements due to the phenomenon of regression to the average, the recommendations guide us towards employing values of ≤ 140/90 mmHg.^3^ Thus, we cannot exclude the possibility that the percentage of hypertensive patients was underestimated in the sample studied.

The average monthly cost of caring for hypertension (R$ 89.90) consumed around 23% of the per-capita income. Most of the direct costs associated with hypertension related to the medications. This is an important conclusion that must be disseminated among the medical community. Almost ¼ of earnings were consumed on the medications, confirming our findings that 89% of patients attributed the low adherence to the high cost of the medications. Curiously, however, in a survey that we made among Brazilian doctors, the cost was the fourth item to be considered in the choice of prescriptions of medications, coming after personal experience, patient characteristics and efficacy.^4^

Another important point of this study concerns the cost-effectiveness relationship, which was more favorable for diuretics and beta-blockers than for angiotensin conversion enzyme inhibitors or calcium channel blockers. In fact, diuretics are still the most prescribed medications in Brazil. In the study among Brazilian doctors cited above, diuretics were the drugs most often mentioned for starting treatments. Nonetheless, the present study shows that only 55% of the patients who were taking diuretics presented an arterial pressure of < 160/95 mmHg. This figure for patients whose pressure was controlled through diuretics may be an overestimate, if the present recommended value of < 140/90 mmHg is considered, a value that is difficult to obtain using monotherapy.^5^ In addition to this, the authors show that hypertension control using monotherapy was more frequently achieved in patients taking calcium channel antagonists (80%) and beta-blockers (71%).

An important question arises at this point, when costeffectiveness studies are considered. In addition to being efficacious in reducing the pressure, diuretics present such low costs that it would be difficult for another drug to be able to present better cost-effectiveness. Nevertheless, although we employ the costeffectiveness rate to measure drug efficacy in the real world, the question that arises is what is most important: spending less or controlling a greater percentage of patients? We believe that, the objective is always to control more and better because the future incidence of heart failure, cerebrovascular disease and myocardial infarction will probably be lower. In addition to this, the causes of the low percentages of control obtained using diuretics must come into the reckoning. With diuretics, the collateral effects and worsening of the quality of life, causing absenteeism from work and additional medical consultations or laboratory examinations, as well as the need for additional medications for treating the collateral effects (potassium supplements, uricosuric agents etc.),^6^ appear to be extremely important aspects.

Consequently, the authors' conclusion must also be viewed from the standpoint of the questions raised above, since they conclude that the treatment of hypertension using diuretics or betablockers was more cost-effective than treatment using angiotensin conversion enzyme inhibitors and calcium channel blockers.
